# IFN‐α treatment may enable discontinuation of TKIs in NK cell‐licensed patients with CML‐CP

**DOI:** 10.1002/jha2.1053

**Published:** 2024-11-26

**Authors:** Hiroshi Ureshino, Kazuharu Kamachi, Keisuke Kidoguchi, Shinya Kimura

**Affiliations:** ^1^ Division of Hematology Respiratory Medicine and Oncology Department of Internal Medicine Faculty of Medicine Saga University Saga Japan

**Keywords:** chronic myeloid leukemia, interferon‐α, *KIR3DL1‐HLA‐Bw* status, NK cell immune response, treatment‐free remission

## Abstract

The magnitude of the natural killer (NK) cell response contributes to the achievement of treatment‐free remission (TFR) in patients with chronic myeloid leukemia (CML) and is regulated by the interaction between killer immunoglobulin‐like receptors (KIRs) on NK cells and human leukocyte antigen (HLA) class I molecules on target cells. The abundant combination between *KIR* and *HLA* through genetic polymorphisms determines the functional diversity of NK cells. We previously reported that *KIR3DL1‐HLA‐Bw* status is associated with achievement of TFR by reflecting NK cell potential. Patients with strong interaction between *KIR3DL1/HLA‐Bw* were identified as having a higher molecular relapse risk, based on the “missing self” hypothesis which suggests that the lack of cognate ligands for KIRs may induce target cell lysis. However, all the patients with strong interaction between *KIR3DL1/HLA‐Bw* who received prior IFN‐α therapy achieved TFR (*p* = 0.007), explained by the “NK cell licensing” concept, whereby NK cells become more functional through the recognition “self” HLA class I molecules by KIRs. NK cell licensing may contribute to the potential efficacy of IFN‐α treatment in patients with CML. We defined high‐risk molecular relapse patients and suggest that *KIR3DL1/HLA‐Bw* status may help detect patients who could benefit from IFN‐α for maintaining TFR.

## 1 INTRODUCTION

Survival outcomes for patients with chronic phase chronic myeloid leukemia (CML‐CP) have markedly improved following the introduction of ABL1 tyrosine kinase inhibitors (TKIs), with most patients no longer dying from CML progression. Achieving treatment‐free remission (TFR) after discontinuing TKI therapy has become a therapeutic goal for patients with CML‐CP; however, the prognostic factors that predict successful TFR achievement remain unclear.

The magnitude of the natural killer (NK) cell response is determined by the interaction between surface molecules on NK cells and their cognate ligands on target cells [1]. The killer immunoglobulin‐like receptor (KIR) is a key NK cell surface molecule involved in the recognition, binding, and/or adhesion to human leukocyte antigen (HLA) class I molecules on target cells. The diverse interactions between KIR and HLA, driven by genetic polymorphisms, determine the functional diversity of NK cells. The strength of the NK cell response has been shown to contribute to the achievement of TFR in patients with CML‐CP [2]. We previously reported that *KIR3DL1‐HLA‐Bw* status is associated with the achievement of TFR by reflecting NK cell‐mediated cancer immunosurveillance potential in patients with CML‐CP [3]. In the POKSTIC study, patients with a strong interaction between *KIR3DL1* and *HLA‐Bw* were identified as having a higher risk of molecular relapse after TKI discontinuation, based on the “missing self” hypothesis (Figure S1A,B), which suggests that NK cells lacking cognate ligands for KIRs may induce target cell lysis [4]. It is necessary to establish alternative treatment strategies for genetically identified high‐risk patients (approximately 40% of patients in the POKSTIC trial) whose impaired NK cell activity may limit their ability to maintain TFR.

Interferon (IFN)‐α combined with TKIs may be a potential alternative treatment strategy for preventing molecular relapse through immune cell activation in high‐risk patients [5]. Indeed, IFN‐α plus TKIs have been shown to increase the long‐lasting mature NK cell fraction in patients with CML [6]. IFN‐α could increase the chance of durable molecular remission [5] and TFR success when it was given in combination with imatinib prior to imatinib discontinuation [7]. Thus, IFN‐α may be an effective treatment in CML, although its use has been limited due to concerns about unacceptable toxicity in general clinical settings.

Another important mechanism regulating NK cells is the process by which NK cells become more functionally mature through the recognition of specific ligands—“self” major histocompatibility complex class I molecules—by KIR, a process known as “NK cell licensing” (Figure S1C,D) [8]. Furthermore, NK cell licensing promotes the expansion of memory NK cells, which has been associated with positive clinical outcomes in antiviral responses [9]. Therefore, NK cell licensing may enhance NK cell responses in immunotherapy settings. We hypothesized that NK cell licensing may contribute to enhancing the effects of IFN‐α treatment in maintaining TFR in patients with CML.

## 2 METHODS

This study was a sub‐study of the POKSTIC trial, an observational study conducted at 18 hospitals in Japan to investigate the association between KIR/HLA genotypes and TFR in CML. The clinical trial was approved by the institutional review board of each participating hospital and is registered with the UMIN Clinical Trials Registry (UMIN000041798). All procedures involving human participants were conducted in accordance with the principles of the Declaration of Helsinki, and all participants provided written informed consent. Allelic genotyping of *KIR* and *HLA* genes was performed as previously described, and the ligands for inhibitory *KIRs* and *KIR2DS1* were identified. We defined three *KIR3DL1/HLA‐Bw* interaction groups to assess the risk of molecular relapse in CML patients: the non‐*KIR3DL1‐Bw* interaction group (lacking *KIR3DL1* or *Bw6* with *KIR3DL1*), the weak *KIR3DL1‐Bw* interaction group (*KIR3DL1* with *Bw4* [*HLA‐A*]), and the strong *KIR3DL1‐Bw* interaction group (*KIR3DL1* with *Bw4‐80Ile* [*HLA‐B*]/*80Thr*), as previously described. Molecular relapse was defined as loss of deep molecular response (DMR) (≥0.01% *BCR::ABL1*mRNA) at two consecutive timepoints or loss of major molecular response (≥0.1% *BCR::ABL1* mRNA) at a single timepoint. All variables affecting TFR were assessed using the Kaplan‒Meier method, differences were analyzed using the log‐rank test and Cox's proportional hazard model. Significant differences between the two groups were established using Mann‒Whitney tests and paired *t*‐tests, with *p* < 0.05 considered significant. All statistical analyses were performed using EZR (Saitama Medical Center, Jichi Medical University).

## 3 RESULTS AND DISCUSSION

Between October 19, 2020, and September 7, 2022, 76 patients enrolled in the POKSTIC trial. The median age was 63 years (interquartile range [IQR]: 49‒70 years); 41 patients were male and 35 were female. Nine patients had been previously treated with IFN‐α, with a median treatment duration of 23 months (range: 6‒37 months), administered a median of 109 months (range: 20‒147 months) before TKI discontinuation. The TKI administered prior to discontinuation was imatinib in 28 cases and dasatinib in 48 cases. A total of 42 out of 76 patients (56.6%; 95% confidence interval [CI]: 47.7%‒66.8% at 6 months) with CML‐CP discontinued TKIs without experiencing molecular relapse, and the median follow‐up time for TFR was 24 months (IQR: 16‒64 months) [3].

**FIGURE 1:**
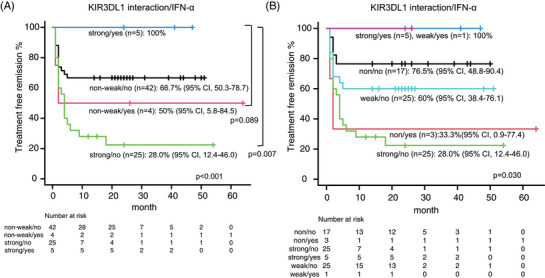
Treatment‐free remission (TFR) according to KIR3DL1‐HLA‐Bw interaction (divided into two groups; non‐weak/strong) and presence or absence of prior interferon (IFN)‐α treatment (A). TFR according to KIR3DL1 and HLA‐Bw interaction (divided into three groups; non/weak/strong) and prior IFN‐α treatment (B).

Patients with prior IFN‐α treatment tended to have a lower risk of molecular relapse (hazard ratio [HR]: 0.406; 95% CI: 0.097‒1.691; *p* = 0.215), indicating that IFN‐α may help some patients achieve TFR, consistent with previous studies on IFN‐α maintenance therapy [7]. The duration of IFN‐α treatment did not affect the achievement of TFR (HR: 0.866; 95% CI: 0.054‒13.95; *p* = 0.919). Patients with a strong interaction between *KIR3DL1* and *HLA‐Bw* showed a high risk of molecular relapse, as previously described [3]. However, notably, all patients with a strong interaction who had received prior IFN‐α treatment achieved TFR (*p* = 0.009, Figure 1A). Furthermore, one patient with a weak interaction between *KIR3DL1* and *HLA‐Bw* who had received prior IFN‐α also maintained TFR (Figure 1B). These findings suggest that the benefits of prior IFN‐α treatment may paradoxically apply only to patients with *KIR3DL1/HLA‐Bw* interaction, which might otherwise interfere with NK cell activation and achievement of TFR, according to the “missing self” hypothesis.

**FIGURE 2:**
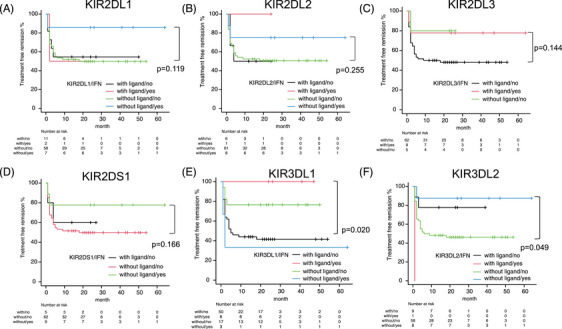
Treatment‐free remission (TFR) according to the presence or absence of cognate ligand for killer immunoglobulin‐like receptor and presence or absence of prior interferon (IFN)‐α treatment. KIR2DL1 (A), KIR2DL2 (B), KIR2DL3 (C), KIR2DS1 (D), KIR3DL1 (E), and KIR3DL2 (F).

Therefore, we investigated five inhibitory and one activating KIR genes, including *KIR2DL1*, *KIR2DL2*, *KIR2DL3*, *KIR2DS1*, *KIR3DL1*, and *KIR3DL2*, to determine whether the presence of matching ligands for these *KIRs* might enhance the effects of IFN‐α, leading to the achievement of TFR. Only patients with *KIR3DL1* and their matching ligands showed positive effects from IFN‐α (*p* = 0.020, Figure 2A‒E). Conversely, patients with *KIR3DL2* without matching ligands also showed positive effects from IFN‐α (*p* = 0.049, Figure 2F). Notably, the patients who benefited from IFN‐α were those with *KIR3DL1* and matching ligands (nos. 24, 27, 36, 59, 62, and 79) and those with *KIR3DL2* without matching ligands (nos. 10, 24, 27, 36, 59, 62, 64, and 79), suggesting that a favorable *KIR/HLA* haplotype may exist.

**FIGURE 3:**
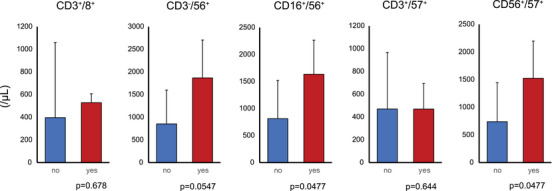
T and natural killer cell counts at dasatinib discontinuation according to presence or absence of prior interferon‐α treatment.

Patients who received prior IFN‐α treatment had significantly higher CD16+CD56+ (median 1561.2 vs. 595.5/µL, *p* = 0.047) and CD56+CD57+ (median 1397.3 vs. 526.2/µL, *p* = 0.047) NK cell fractions at dasatinib discontinuation compared to those who did not, indicating that IFN‐α may promote mature NK cell differentiation (Figure 3) [10].

Notably, although the IFN‐α treatment was administered approximately 9 years earlier, the effects persisted long‐term, as previously reported. One possible explanation is the presence of memory NK cells, which expand through NK cell education via KIR/HLA interactions during viral infections and exhibit enhanced antiviral cytotoxicity [9]. We speculate that a similar phenomenon may have occurred in patients with CML treated with IFN‐α.

Although dasatinib is known to increase NK cells, all patients who had their T/NK cell populations evaluated in the POKSTIC study were receiving dasatinib [11, 12] (T/NK cell fractions were not assessed in patients receiving imatinib). Therefore, the differences observed in NK cell counts could be attributed to IFN‐α effects. Notably, the NK cell expansion induced by dasatinib was transient, with NK cell fractions decreasing 3 months after dasatinib discontinuation (Figure S2). In contrast, NK cell expansion induced by IFN‐α persisted long‐term, suggesting that IFN‐α may be a suitable maintenance therapy for TFR.

The *KIR3DL1* gene exhibits extensive allelic polymorphism, and its protein product interacts with its cognate ligand, the HLA‐Bw4 epitope, to regulate NK cell immune responses. Based on the “missing self” hypothesis [4], the combination of *KIR3DL1* and *HLA‐Bw4* has been shown to influence clinical outcomes in patients with viral infections [13] and malignancies [3]. In line with this hypothesis, we previously reported that *KIR3DL1‐HLA‐Bw4* status may predict TFR outcomes, where patients with strong interactions between *KIR3DL1* and *HLA‐Bw4* were found to have unfavorable TFR prognosis.

Interestingly, positive effects of IFN‐α were observed in patients who possessed *KIR3DL1* with matching ligands, potentially reflecting “NK cell licensing” effects (Figure S1C,D). IFN‐α promotes memory‐cell differentiation in NK cells [14], leading to enhanced NK cell immune responses in adaptive immunotherapy. Since our data suggested that educated NK cells play a crucial role in IFN‐α therapy for CML, we recommend IFN‐α maintenance therapy for TFR in patients who possess *KIR3DL1* with its cognate ligand. Conversely, for patients lacking *KIR3DL1/HLA‐Bw* interaction, long‐term TKI therapy (over 5 years) is recommended to maintain DMR for achieving TFR [15].

Based on *KIR3DL1/HLA‐Bw* status, we have identified high‐risk patients for molecular relapse and potential candidates who may benefit from IFN‐α maintenance therapy for TFR. We propose the above consolidation strategy as an optimal approach. Future prospective studies are needed to elucidate the role of “NK cell education” in “IFN‐α treatment.”

## AUTHOR CONTRIBUTIONS

Hiroshi Ureshino and Shinya Kimura made substantial contributions to study conception, design and data analysis, and interpretation. Shinya Kimura enrolled patients and collected data. Hiroshi Ureshino, Kazuharu Kamachi, Keisuke Kidoguchi and Shinya Kimura wrote the paper. Hiroshi Ureshino and Shinya Kimura critically reviewed the drafts. All authors approved the final version.

## CONFLICT OF INTEREST STATEMENT

Shinya Kimura received honoraria from Bristol‐Myers‐Squibb, Novartis, Pfizer, and Otsuka Pharmaceuticals, and research funding from Bristol‐Myers‐Squibb, Pfizer, and Ohara Pharmaceuticals. The remaining authors declare they have no conflicts of interest.

## ETHICS STATEMENT

This study was approved by the institutional review board of each participating hospital and performed in accordance with the ethical principles in the Declaration of Helsinki.

## PATIENT CONSENT STATEMENT

All patients provided written informed consent prior to enrollment in the study.

## CLINICAL TRIAL REGISTRATION

The trial was registered at UMIN000041798.

## REFERENCES

## Supporting information



Supporting Information

## Data Availability

All data are available from the corresponding author upon reasonable request.
