# Supplementary material for: IFN‐α treatment may enable discontinuation of TKIs in NK cell‐licensed patients with CML‐CP
Source: EJHaem. 2024 Nov 26;5(6):1278–82. doi: 10.1002/jha2.1053 (PMC11647684; doi:10.1002/jha2.1053)
Supplement: Supplementary file 1 — Supporting Information [file JHA2-5-1278-s001.pdf]

Figure S1

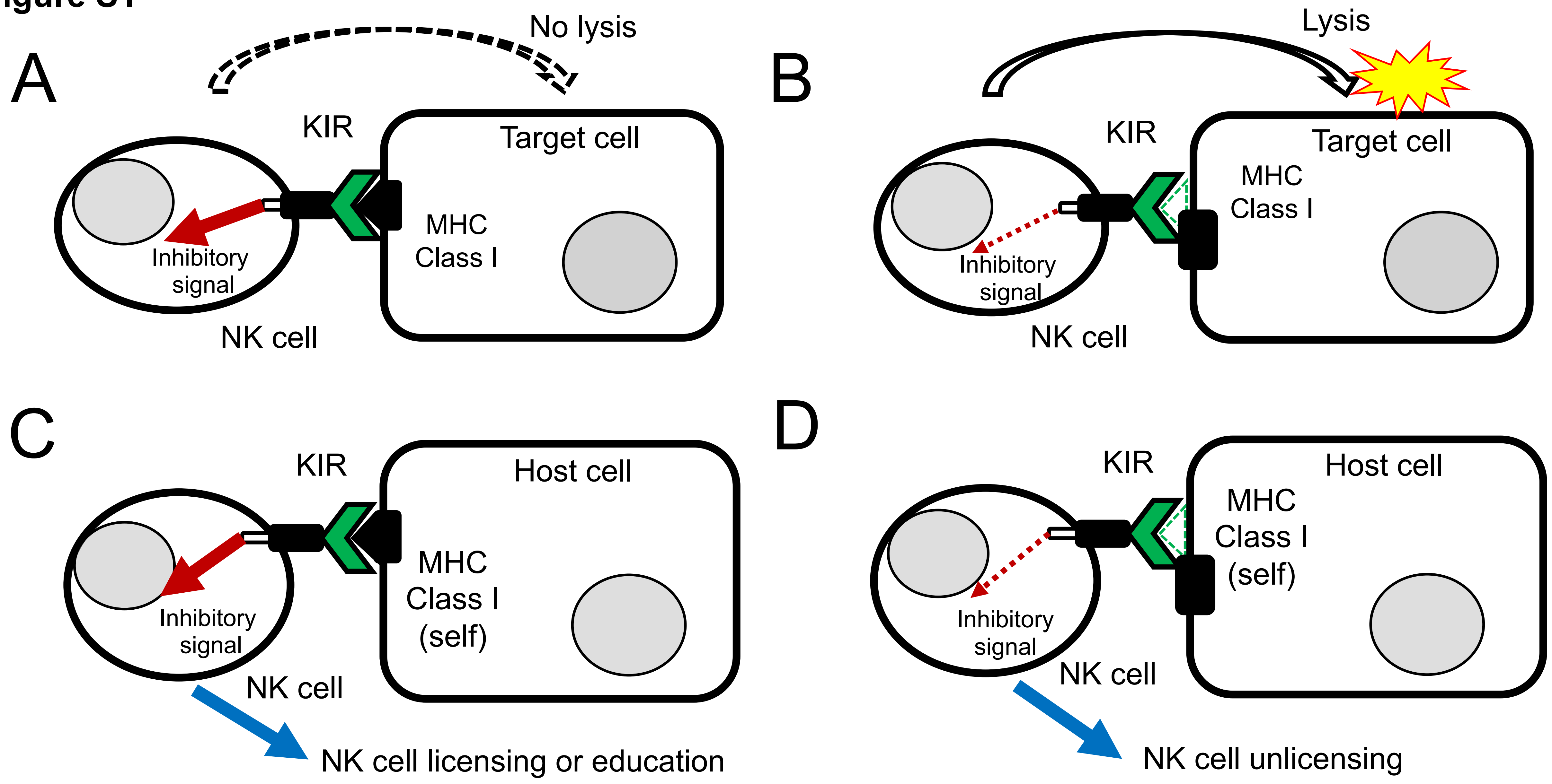

Figure S1. Missing self hypothesis (A, B) and natural killer (NK) cell licensing (C, D) of NK cells.

Figure S2

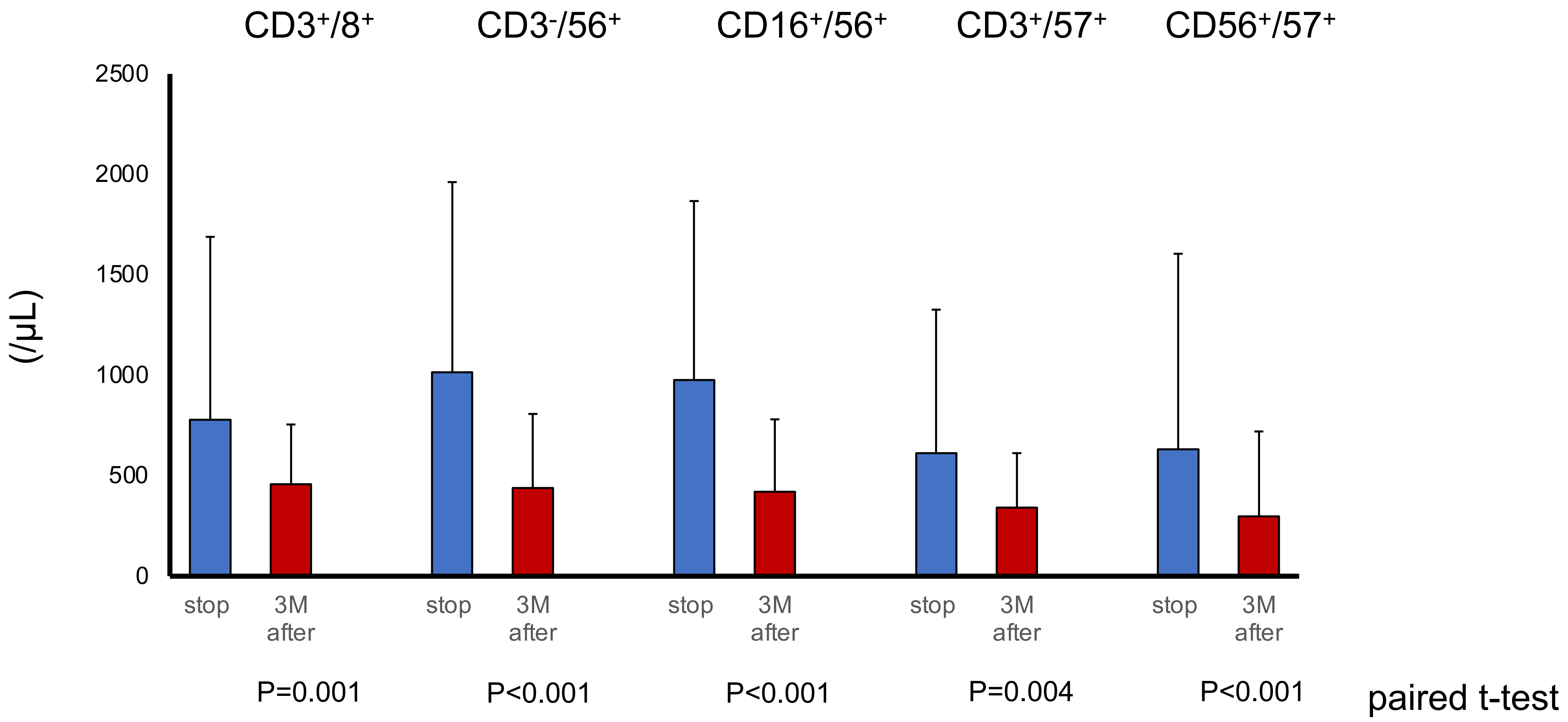

Figure S2. T and natural killer cell count at dasatinib discontinuation and three months after dasatinib discontunuation
